# Association of subsequent treated shockable rhythm with outcomes after paediatric out-of-hospital cardiac arrests: A nationwide, population-based observational study

**DOI:** 10.1016/j.resplu.2021.100181

**Published:** 2021-11-09

**Authors:** Yoshikazu Goto, Akira Funada, Tetsuo Maeda, Yumiko Goto

**Affiliations:** a Department of Emergency and Critical Care Medicine, Kanazawa University Hospital, Takaramachi 13-1, Kanazawa 920-8640, Japan; b Department of Cardiology, Osaka Saiseikai Senri Hospital, Tukumodai, 1-1-6, Suita 565-0862, Japan; c Department of Cardiology, Yawata Medical Center, Yawata I 12-7, Komatsu 923-8551, Japan

**Keywords:** Out-of-hospital cardiac arrest, Resuscitation, Children, Outcomes, Non-shockable rhythm, Rhythm conversion

## Abstract

**Aim:**

Among patients with paediatric out-of-hospital cardiac arrests (OHCAs), most have an initial non-shockable rhythm with poor outcomes. There is a subset who developed shockable rhythms. This study aimed to investigate the association between subsequent shock delivery and outcomes after paediatric OHCAs.

**Methods:**

We analysed records of 19,095 children (aged <18 years) with OHCA and initial non-shockable rhythm. Data were obtained from a Japanese nationwide database for 13 years (2005–2017). The primary outcome measure was 1-month neurologically intact survival, defined as cerebral performance category 1–2.

**Results:**

Among patients with pulseless electrical activity (PEA, n = 3,326), there was no significant difference between those with subsequent treated shockable rhythm (10.0% [11/109]) and those with sustained non-shockable rhythm (6.0% [192/3,217], p = 0.10) with respect to the neurologically intact survival rate. Among asystole patients (n = 15,769), the neurologically intact survival rate was significantly higher in the subsequent treated shockable rhythm group (4.4% [10/227]) than in the sustained non-shockable rhythm group (0.7% [106/15,542], p < 0.0001). Subsequent treated shockable rhythm with a shock delivery time (time from emergency medical services [EMS]-initiated cardiopulmonary resuscitation [CPR] to shock delivery) ≤9 min was associated with increased odds of neurologically intact survival compared with sustained non-shockable rhythm (PEA, adjusted odds ratio, 2.45 [95% confidence interval, 1.16–5.16], p = 0.018; asystole, 9.77 [4.2–22.5], p < 0.0001).

**Conclusion:**

After paediatric OHCAs, subsequent treated shockable rhythm was associated with increased odds of 1-month neurologically intact survival regardless of whether the initial rhythm was PEA or asystole, only when the shock was delivered ≤9 min of EMS-initiated CPR.

## Introduction

Early defibrillation is a key component in the chain of survival after paediatric out-of-hospital cardiac arrest (OHCA), especially after initial shockable rhythms (ventricular fibrillation [VF] or pulseless ventricular tachycardia).1., 2., 3. Children who experienced OHCA with initial shockable rhythms have better favourable outcomes than those with initial non-shockable rhythm (pulseless electrical activity [PEA] or asystole).2., 4. However, the proportion of initial shockable rhythms in children with OHCA is very low, ranging from 4.9% to 10.0%,5., 6., 7., 8. with a higher incidence among adolescents. Among children who received defibrillation after OHCA, 32.7–34.9% initially presented with a non-shockable rhythm.6., 7. Rhythm conversion to shockable rhythm is of clinical importance.5., 6., 7. An earlier meta-analysis of adults with OHCA^9^ revealed that shock delivery following conversion from non-shockable to shockable rhythm was associated with better outcomes, depending on the type of initial rhythm and time of rhythm conversion. Rhythm conversion from asystole, but not PEA, was associated with the prehospital return of spontaneous circulation (ROSC) and survival to hospital discharge. Moreover, earlier shockable rhythm conversions were associated with higher odds of 1-month favourable neurological outcomes compared with those occurring later during cardiopulmonary resuscitation (CPR) in adults.^9^ A previous observational study of paediatric OHCAs revealed that subsequent treated shockable rhythm was associated with improved neurologically intact survival compared with sustained non-shockable rhythm.^10^ However, subgroups with initial non-shockable rhythms have not been previously investigated in a paediatric population.

Thus, this study aimed to elucidate the association of subsequent treated shockable rhythm with outcomes based on the type of initial non-shockable rhythm and CPR duration until shock delivery in children with OHCA using the Japanese nationwide registry data.

## Methods

### Study design and setting

This investigation was a nationwide population-based observational study in Japan and included all paediatric patients (aged <18 years) who experienced OHCA and were resuscitated by emergency medical services (EMS) personnel between 1 January 2005 and 31 December 2017. Patients were excluded if they (1) were aged ≥18 years, (2) did not receive resuscitation from EMS personnel, (3) had initial shockable rhythms, or (4) had unknown time variables, outcomes, or age.

The Fire and Disaster Management Agency (FDMA) in Japan supervises the nationwide EMS system, while local fire stations operate the local EMS systems. As of 2017, the country has 732 fire departments and 5140 ambulance teams.^11^ During the study period, all EMS personnel performed CPR according to the Japanese guidelines.12., 13., 14. Moreover, emergency lifesaving technicians who were EMS personnel used several other resuscitation techniques such as automated external defibrillators (AEDs), airway adjuncts, peripheral intravenous catheters, and administration of Ringer’s lactate solution.^11^ In the field, only specially trained emergency lifesaving technicians, upon receiving instructions from an online physician, are permitted to insert a tracheal tube and administer intravenous adrenaline (epinephrine).^11^ EMS personnel in Japan are legally prohibited from terminating resuscitation in the field. Therefore, most OHCA patients receive CPR from EMS personnel before being transported to a hospital with the exception of definitive situations, such as decapitation, incineration, decomposition, rigor mortis, or dependent cyanosis. When EMS providers arrive at a scene, initiation of CPR and initial rhythm assessment through an AED are generally performed. An AED delivers a shock only when it detects a shockable rhythm. When the initial non-shockable rhythm is identified, rhythm analysis is performed every 2 min by AED during CPR.

### Data collection and quality control

In January 2005, the FDMA launched an ongoing, prospective, population-based observational study involving patients with OHCA who had received resuscitation from EMS personnel in Japan.^11^ EMS personnel from each centre recorded the data of patients with OHCA using a Utstein-style template with the cooperation of the physician-in-charge.15., 16. All the data were transferred and stored in the nationwide database developed by the FDMA for public use. The FDMA granted permission to access the database and provided anonymous data for our analysis. The main variables included in the dataset were sex, age, aetiology of arrest, initially identified cardiac rhythm, bystander-witnessed status, type of bystander CPR, time of collapse recognition, time of emergency call, time of vehicle arrival at the scene, time of CPR initiation by EMS, prehospital ROSC, 1-month survival, and 1-month neurological outcomes. The aetiology of arrest was presumed to be cardiac unless evidence suggested traumatic causes (i.e., injury from a traffic accident, fall, accidental hypothermia, hanging, drowning, drug overdose/poisoning, or asphyxia) or other noncardiac causes such as respiratory disease, cerebrovascular disease, or malignant tumours. The physicians in charge and EMS personnel attempted to determine the origin of the arrest. Neurological outcomes were defined using the cerebral performance category (CPC) scale (category 1, good cerebral performance; category 2, moderate cerebral disability; category 3, severe cerebral disability; category 4, coma, or vegetative state; and category 5, death).^16^ CPC categorisation was determined by the physician-in-charge 1 month after cardiac arrest. Information on bystander interventions was obtained by EMS personnel who interviewed bystanders before leaving the scene. All data were electronically recorded by EMS personnel and/or EMS centre. The time data were recorded electrically on a recording medium according to the times on the clock used by the EMS system that responded to the call. In particular, the time of the first shock delivery was validated using data from defibrillator recordings.

### Study endpoints

The primary outcome measure was 1-month neurologically intact survival, defined as a CPC score of 1 or 2. The secondary outcome measures were prehospital ROSC and 1-month survival after OHCA.

### Statistical analysis

We used the Kruskal–Wallis test followed by the Dunn’s post hoc test to analyse continuous variables. The chi-square test and univariate logistic regression analyses were performed to compare the characteristics and outcomes of the categorical variables. Furthermore, we analysed multivariable logistic regression models to clarify the relationship between subsequent treated shockable rhythm and outcomes. In the multivariable logistic regression analyses, potential prehospital confounders for the analytical model were selected based on biological plausibility and data from previous studies, which were as follows: sex, age, witnessed status, cause of arrest, shock delivery time (defined as CPR duration from EMS-initiated CPR to first shock delivery), bystander-initiated CPR, advanced airway management, epinephrine administration, and call-to-response time (time from emergency call to EMS arrival at the patient’s location). We classified prehospital shock delivery variables into three categories (no-shock delivery, shock delivery with delivery time of ≤ 9 min, and shock delivery with delivery time of >9 min), referring to median values of overall shock delivery time (9 min, interquartile range, 5 min to 14 min), for the multivariable logistic regression analytic model.

Continuous variables are expressed as medians and interquartile ranges or as means and standard deviations, whereas categorical variables are expressed as percentages. As an estimate of effect size and variability, we reported odds ratios (ORs) with 95% confidence intervals (CIs). All statistical analyses were performed using the JMP statistical package, version 15-Pro (SAS Institute Inc., Cary, NC, USA). All reported tests were two tailed, and a *P* value <0.05 was considered significant.

## Results

**Fig. 1:**
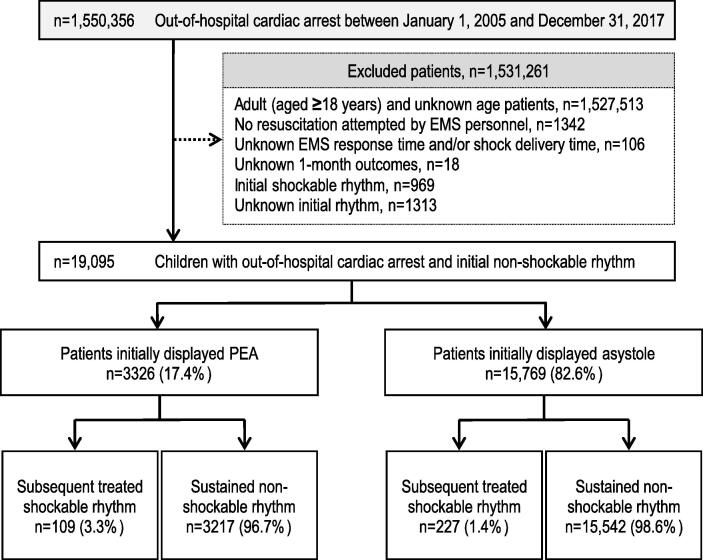
Flowchart of patient inclusion criteria. EMS: emergency medical services, PEA: pulseless electrical activity.

Details of attempted resuscitations performed for 1,550,356 OHCA patients between 2005 and 2017 are documented in the database. Fig. 1 presents the inclusion and exclusion criteria of the study. Patients with missing data (n = 1437) were excluded from the analysis: unknown EMS response and/or shock delivery time (n = 106), unknown 1-month outcomes (n = 18), and unknown initial rhythm (n = 1313). Finally, 19,095 paediatric patients (1.2% of registered patients) with OHCA were eligible. Based on the type of initial non-shockable rhythm, we divided patients with OHCA into patients initially displayed PEA (n = 3326, 17.4%) and patients initially displayed asystole (n = 15,769, 82.6%). Furthermore, the two cohorts were further subdivided according to the presence of rhythm conversion: subsequent treated shockable rhythm and sustained non-shockable rhythm groups. Patients with an initial non-shockable rhythm who converted to shockable rhythms were identified by shocks delivered later during resuscitation. These were assigned to the subsequent treated shockable rhythm group. Thus, the delivery of subsequent shocks was used as a surrogate marker for conversion to a shockable rhythm. Conversely, the sustained non-shockable rhythm group comprised patients who did not receive any shocks during resuscitation.

The baseline characteristics of the patients according to the type of initial non-shockable rhythm are shown in Table 1. Compared with patients with initial asystole, patients with initial PEA were older and had higher proportions of witnessed arrest and epinephrine administration, shorter shock delivery time, and lower proportions of presumed cardiac aetiology and bystander CPR. The proportion of patients with subsequent treated shockable rhythm and those with favourable outcomes was significantly higher in the PEA group than in the asystole cohort. In the initial PEA cohort, patients with subsequent treated shockable rhythm were older and had higher rates of cardiac aetiology compared with patients with sustained non-shockable rhythm (Table 2). In the cohort of patients with initial asystole, those with subsequent treated shockable rhythm were older and had higher rates of witnessed arrest, advanced airway management, epinephrine administration compared with patients with sustained non-shockable rhythm (Table 3).

**Table 1:** Baseline characteristics of patients according to initial non-shockable rhythm.

	PEA	Asystole	*P* value
Patients, n	3326	15,769	
Age, years			
Median (IQR)	3 (0–13)	1 (0–11)	<0.001
Male, n (%)	2023 (60.8)	9588 (60.8)	0.98
Presumed cardiac aetiology, n (%)	910 (27.4)	4923 (31.2)	<0.001
Witnessed cardiac arrest, n (%)	1982 (59.6)	3344 (21.1)	<0.001
Bystander CPR, n (%)	1594 (47.9)	8660 (54.9)	<0.001
Advanced airway management, n (%)	533 (16.0)	2392 (15.2)	0.21
Epinephrine administration, n (%)	143 (4.3)	356 (2.3)	<0.001
EMS response time, min			
Median (IQR)	8 (6–10)	8 (6–10)	<0.001
Call-to-hospital arrival time, min			
Median (IQR)	27(21–35)	27(21–34)	<0.05
Subsequent treated shockable rhythm, n (%)	109 (3.3)	227 (1.4)	<0.001
Shock delivery time, min, n = 336			
Median (IQR)	8 (5–12)	10 (6–16)	<0.01
Prehospital return of spontaneous circulation, n (%)	433 (13.0)	371 (2.4)	<0.001
1-month outcomes			
Survival, n (%)	651 (19.6)	910 (5.8)	<0.001
CPC 1 or 2, n (%)	203 (6.1)	116 (0.7)	<0.001

CPC, Cerebral Performance Category; CPR, cardiopulmonary resuscitation; EMS, emergency medical services; IQR, interquartile range; PEA, pulseless electrical activity.

**Table 2:** Baseline characteristics of patients with initial pulseless electrical activity according to rhythm conversion status.

			Subsequent treated shockable rhythm	Sustained non-shockable rhythm	*P* value
Patients, n			109	3217	
Age, years					
	Median (IQR)		11 (6–15)	3 (0–13)	<0.0001
Male, n (%)			64 (58.7)	1959 (60.9)	0.69
Cardiac aetiology, n (%)			61 (56.0)	849 (26.4)	<0.0001
Witnessed cardiac arrest, n (%)			73 (67.0)	1909 (59.3)	0.11
Bystander CPR, n (%)			57 (52.3)	1537 (47.8)	0.38
Advanced airway management, n (%)			84 (77.1)	2709 (84.2)	0.06
Epinephrine administration, n (%)			9 (8.3)	134 (4.2)	0.05
EMS response time, min					
	Median (IQR)		8 (6–9)	8 (6–10)	0.83
Call-to-hospital arrival time, min					
	Median (IQR)		29 (23–37)	27 (21–35)	0.05

CPR, cardiopulmonary resuscitation; EMS, emergency medical services; IQR, interquartile range.

**Table 3:** Baseline characteristics of patients with initial asystole according to rhythm conversion status.

			Subsequent treated shockable rhythm	Sustained non-shockable rhythm	*P* value
Patients, n			227	15,542	
Age, years					
	Median (IQR)		13 (5–16)	1 (0–11)	<0.0001
Male, n (%)			147 (64.8)	9441 (60.8)	0.24
Cardiac ethology, n (%)			72 (31.7)	4851 (31.2)	0.89
Witnessed cardiac arrest, n (%)			82 (36.1)	3262 (21.0)	<0.0001
Bystander CPR, n (%)			111 (48.9)	8549 (55.0)	0.07
Advanced airway management, n (%)			73 (32.2)	2319 (14.9)	<0.0001
Epinephrine administration, n (%)			32 (14.1)	324 (2.1)	<0.0001
EMS response time, min					
	Median (IQR)		8 (7–11)	8 (6–10)	<0.05
Call-to-hospital arrival time, min					
	Median (IQR)		32 (25–39)	27 (21–34)	<0.0001

CPR, cardiopulmonary resuscitation; EMS, emergency medical services; IQR, interquartile range.

**Fig. 2:**
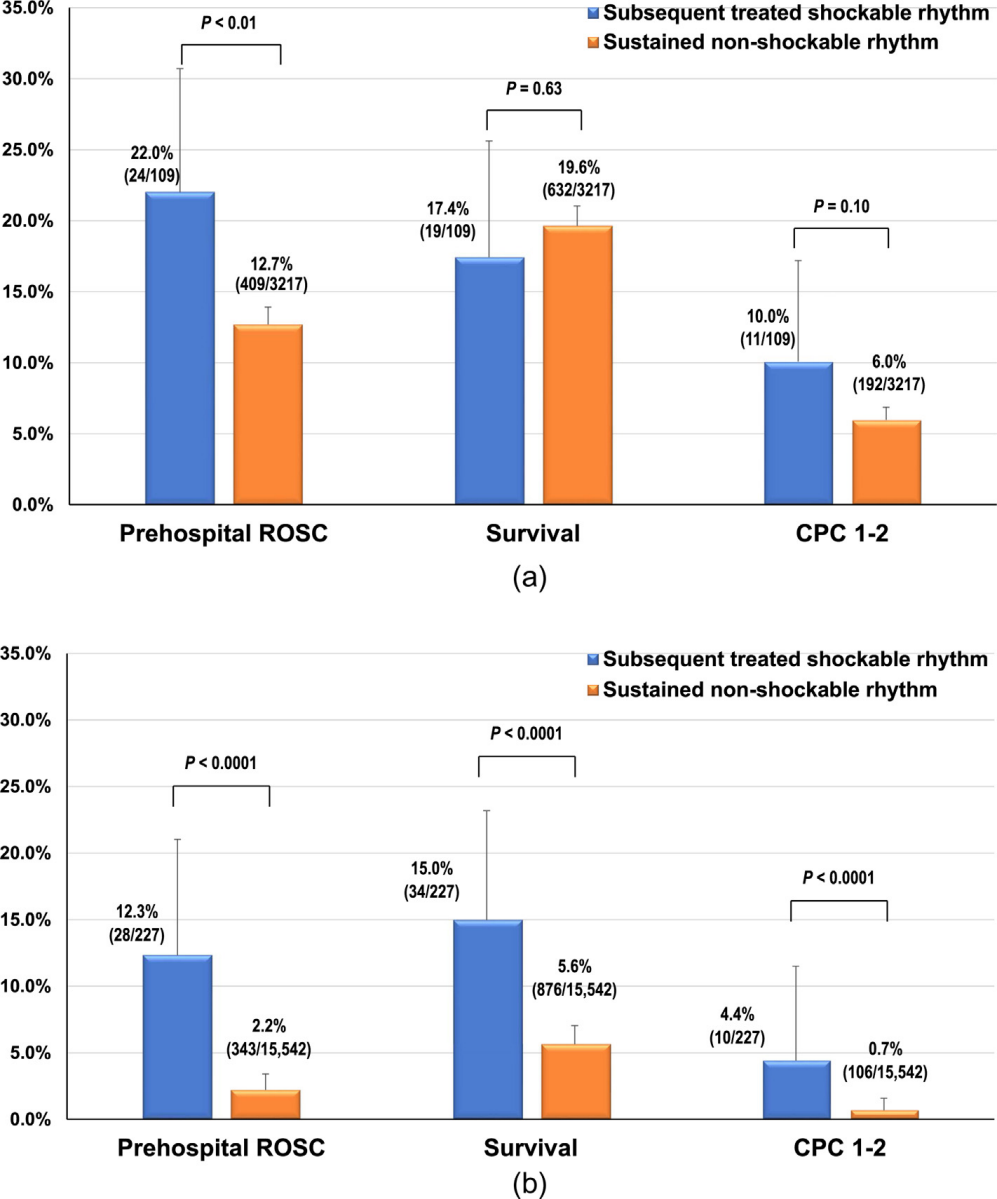
Unadjusted outcomes by initial non-shockable rhythm. (A) Pulseless electrical activity. (B) Asystole. CPC, cerebral performance category; ROSC, return of spontaneous circulation.

The results of the comparison of unadjusted outcomes between the two groups by the initial rhythm are shown in Fig. 2. In the initial PEA cohort, the rate of prehospital ROSC was significantly higher in the subsequent treated shockable rhythm group than in the sustained non-shockable rhythm group. However, there were no significant differences between the two groups with respect to the rates of 1-month survival and 1-month CPC 1–2 (Fig. 2**A**). In the initial asystole cohort, the rates of prehospital ROSC, 1-month survival, and 1-month CPC 1–2 were significantly higher in the subsequent treated shockable rhythm group than in the sustained non-shockable rhythm group (Fig. 2**B**).

**Fig. 3:**
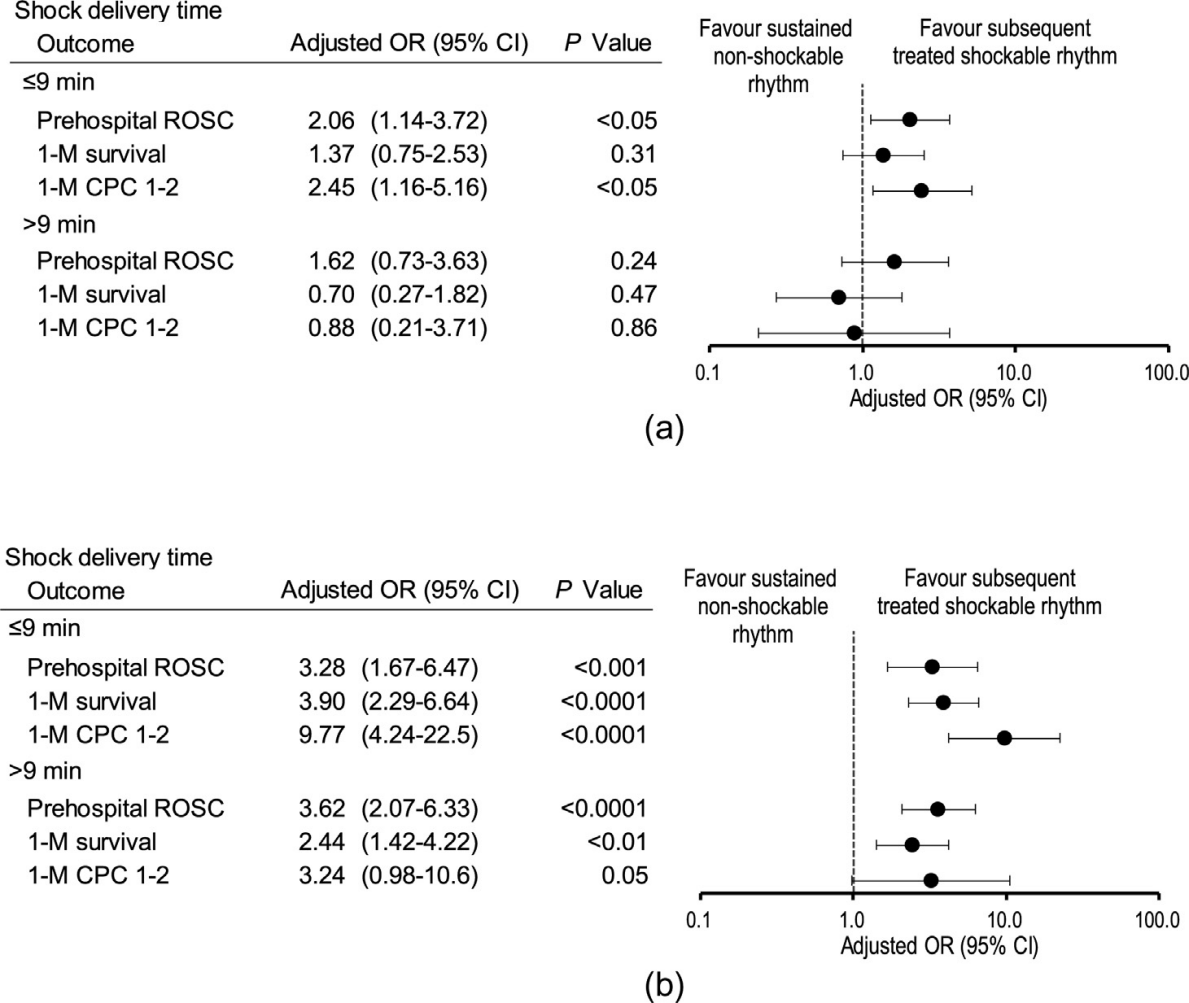
Adjusted odds ratios of subsequent treated shockable rhythm by shock delivery time compared with no shock delivery (sustained non-shockable rhythm). (A) Pulseless electrical activity. (B) Asystole. CI, confidence interval; CPC, cerebral performance category; OR, odds ratio; ROSC, return of spontaneous circulation.

Adjusted odds ratios of subsequent treated shockable rhythm by shock delivery time compared with no shock delivery (sustained non-shockable rhythm) are shown in Fig. 3. In the initial PEA cohort, subsequent treated shockable rhythm with a shock delivery time of ≤9 min was associated with increased odds of prehospital ROSC and 1-month CPC 1–2 compared with sustained non-shockable rhythm, but not 1-month survival. There were no significant differences between the two groups with respect to outcomes when the shock delivery time was >9 min (Fig. 3**A**). In the initial asystole cohort, subsequent treated shockable rhythm was associated with increased odds of prehospital ROSC and 1-month survival compared with sustained non-shockable rhythm regardless of the shock delivery time (Fig. 3**B**). Moreover, subsequent treated shockable rhythm was associated with increased odds of 1-month CPC 1–2 when the shock delivery time was ≤9 min but not >9 min.

## Discussion

This nationwide population-based observational study in Japan demonstrated that compared with sustained non-shockable rhythm, subsequent treated shockable rhythm with a shock delivery time of ≤9 min was associated with increased odds of 1-month neurologically intact survival regardless of whether the initial rhythm was PEA or asystole. Even when shock delivery time was >9 min, subsequent treated shockable rhythm was associated with increased odds of prehospital ROSC and 1-month survival compared with sustained non-shockable rhythm in patients with initial asystole. To the best of our knowledge, this is the first and largest cohort study to show the better association of subsequent treated shockable rhythm with meaningful outcomes and shock delivery time stratified by the type of initial non-shockable rhythm after paediatric OHCA.

Conversion from a non-shockable rhythm to shockable rhythm has been shown to be associated with better outcomes in adult patients with OHCA compared with sustained non-shockable rhythm when rhythm conversion is achieved earlier during CPR.9., 17., 18. To our knowledge, only one study has thus far analysed data on shockable rhythm conversion and outcomes stratified by rhythm conversion time among paediatric OHCAs.^10^ Goto et al. showed that subsequent treated shockable rhythm was associated with improved 1-month survival with favourable neurological outcomes in children with initial non-shockable rhythms after OHCA.^10^ They demonstrated that 1-month survival and 1-month neurologically intact survival decreased as the shock delivery time increased. However, they did not elucidate the association of subsequent treated shockable rhythm with outcome stratified by initial PEA or asystole and shock delivery time in that study. In the present study, we demonstrated that shock delivery time is a key factor that has an impact on the outcomes of children with initial non-shockable rhythm regardless of initial PEA or asystole.

In adult patients with OHCA, Zhang et al.^19^ showed that the association between shockable rhythm conversion and favourable functional outcomes at hospital discharge was stronger among those with initial asystole (adjusted OR, 4.28; 95% CI, 2.32–7.89, compared with no shock delivery) than among those with initial PEA (adjusted OR, 2.26; 95% CI, 1.37–3.75, compared with no shock delivery) when shock was delivered <10 min after EMS-initiated CPR. Moreover, they found that shockable rhythm conversion after initial asystole was associated with favourable neurological outcomes only when rhythm conversion occurred within 15 min of EMS-initiated CPR. However, in patients with initial PEA, shockable rhythm conversion after initial asystole was associated with favourable neurological outcomes only when rhythm conversion occurred within 10 min of CPR. In the present study, the same tendency was observed in 1-month CPC 1–2 among two non-shockable rhythm cohorts when shock delivery time was ≤9 min (adjusted OR, 9.77, for the asystole and 2.45, for the initial PEA, compared with no shock delivery). Furthermore, since subsequent shock delivery had favourable outcomes with prehospital ROSC and 1-moth survival only after initial systole but not after an initial PEA when shock delivery time was >9 min, the initial asystole cohort was predominant over the initial PEA cohort with respect to the effect of conversion to shockable rhythm on outcomes (Figs. 3A and 3B). However, the unadjusted rates of overall 1-month survival and neurologically intact survival were considerably lower in the initial asystole cohort than in the PEA cohort (Table 1, 0.7% vs. 6.1%, p<0.001). These results imply that electrical shocks may no longer bring survival or functional outcome benefits when rhythm conversions occur beyond certain time thresholds, which may be different in the initial PEA or initial asystole. Conceivably, the arrested heart had entered a so-called “metabolic phase” where there was irreversible ischaemic damage, and the heart muscles had become more susceptible to reperfusion injury.^20^ In these scenarios, high quality continued chest compressions with rescue breaths in addition to the administration of adrenaline may be preferable to electrical defibrillation attempts.

The strength of this present study is that it is a nationwide population-based observational study conducted in Japan for 13 years with a large sample size. However, this study had several limitations. First, we could not exclude patients who received shocks for unrecognised initial shockable rhythms or for incorrect indications attributed to electrical misreading, which could result in the overestimation of favourable outcomes. Precise data regarding device-related and operator/circumstance related errors were not obtained. A previous study showed that errors associated with AED use were rare (4%).^21^ Second, as we included only patients with initial non-shockable rhythm who had a subsequent shockable rhythm that was treated with shock delivery in the present study, there was a possibility that we excluded some patients who developed a subsequent shockable rhythm who never received a shock. Moreover, we did not include patients who received subsequent shocks after their arrival at the hospital. If a considerable number of patients were excluded, favourable outcomes in our study population would be falsely high. Third, although we used a uniform data collection procedure, a large sample size, and a population-based design, we could not exclude the possibility of uncontrolled confounders such as pre-existing comorbidities, location of the arrest, quality of bystander CPR, and in-hospital treatments because the study was retrospective and observational. Particularly, the presence of therapeutic hypothermia after cardiac arrest may have influenced the favourable neurological outcomes.^22^ Fourth, as with all epidemiological studies, selection bias may have occurred, and the data may have lacked integrity and validity. Therefore, a prospective study design may be preferable to minimise those biases. Finally, the relevance of our results to other communities with different emergency care systems and protocols remains unknown. The generalisation of our findings to other populations may be limited. Therefore, similar analyses in other countries are required to validate our results.

## Conclusion

After paediatric OHCAs, subsequent treated shockable rhythm was associated with increased odds of 1-month neurologically intact survival regardless of whether the initial rhythm was PEA or asystole, only when the shock was delivered ≤9 min of EMS-initiated CPR. Even when shock was delivered >9 min, subsequent shock was associated with increased odds of prehospital ROSC and 1-month survival compared with sustained non-shockable rhythm in patients with initial asystole.

## Ethical approval

The study was approved by the institutional review board of Kanazawa University (No. 1263). The requirement for written informed consent was waived because the study used anonymised data.

## CRediT authorship contribution statement

**Yoshikazu Goto:** Conceptualization, Formal analysis. **Akira Funada:** Data curation, Writing – review & editing. **Tetsuo Maeda:** Data curation. **Yumiko Goto:** Data curation, Formal analysis, Writing – review & editing.
